# Trends in vascular access among patients on hemodialysis; a nationwide survey from Egypt

**DOI:** 10.1186/s12882-025-04296-9

**Published:** 2025-07-08

**Authors:** Eman Nagy, Karem Salem, Mostafa Abdelsalam, Rasha Shemies, Nehal Elshabrawy, Ahmed M. Abd Elwahab, Alaa Sabry, Alaa A. Elsawi, Ahmed Albeyaly, Hamed Eleraky, Hala Mahmoud, Mohamed Elsayed, Nayel Zaki, Ahmed Noureldin, Ahmed Megahed, Hassan Foula, Basma Sultan, Mohamed Hamed Brawy, Walaa H. Ibrahim, Alshaimaa S. Mubarak, Bishoy Tanagho, Aber-Halim Attallah, Mohamed Ellawag, Hazem Abo Shousha, Emad Samaan

**Affiliations:** 1 Mansoura Nephrology and Dialysis Unit, Mansoura Faculty of Medicine, Dakahlia, Egypt; 2 Internal Medicine Department, Al-Fayoum Faculty of Medicine, Al-Fayoum, Egypt; 3 Dakahlia Health Directorate for Hemodialysis Units, National Health Service, Dakahlia, Egypt; 4 Mansoura International Hospital, National Health Service, Dakahlia, Egypt; 5 Aswan Faculty of Medicine, Aswan University Hospitals, Aswan, Egypt; 6 Alexandria Faculty of Medicine, Alexandria University Hospital, Alexandria, Egypt; 7 Internal Medicine Department, Sohag Faculty of Medicine, Sohag, Egypt; 8 Internal Medicine Department, Portsaid Faculty of Medicine, Portsaid, Egypt; 9 Nephrology Department, Qabbary Specialized Hospital, Alexandria, Egypt; 10 Internal Medicine Department, Suez Canal faculty of Medicine, Ismailia, Egypt; 11 Hemodialysis Unit, Obour Health Insurance Hospital, Kafr El Shiekh, Egypt; 12 Internal Medicine Department, Assiut faculty of Medicine, Assiut, Egypt; 13 Saint Mark Kidney Center, Cairo, Egypt; 14 Internal Medicine Department, Ain Shams Faculty of Medicine, Cairo, Egypt; 15 Al-Fayoum General Hospital, National Health Service, Al-Fayoum, Egypt; 16 Al-Amerya General Hospital, Alexandira, Egypt

**Keywords:** Arteriovenous fistula, Hemodialysis, Tunneled lines, Non-tunneled lines

## Abstract

**Problem statement:**

Vascular Access (VA) in hemodialysis (HD) patients is vital for treatment efficiency and is influenced by Egypt’s healthcare system and socioeconomic factors. It is a complex issue, shaped by both challenges and opportunities within the nation’s healthcare infrastructure.

**Aim:**

To examine trends in VA use and associated characteristics in patients on HD based on data from a nationwide survey in Egypt.

**Methods:**

This cross-sectional study targeted patients on maintenance hemodialysis across Egypt, using stratified cluster sampling from 11 representative governorates. Medical personnel collected data using a structured electronic Google Form questionnaire, which gathered data on patient demographics, clinical details, VA creation and complications, and healthcare access.

**Results:**

The study included 3,582 chronic HD patients. Data were collected over a one-year period from May 2023 to May 2024. An arteriovenous fistula (AVF) was the initial VA for 669 patients (18.7%), while a temporary catheter was used as the initial access in 2,861 patients (79.9%). AVF thrombosis was the primary cause of AVF failure, occurring in 69.7% of cases. Pre-HD VA creation was associated with substantially better fistula maturation, fewer VA numbers, and lower VA-related complications.

**Conclusion:**

Significant regional and sociodemographic variations in VA practices were observed across Egypt. The findings revealed persistent reliance on temporary catheters at HD initiation, with encouraging but limited progress in pre-HD AVF planning. These trends underscore the need for early referral strategies and targeted interventions to optimize vascular access outcomes nationwide.

**Supplementary Information:**

The online version contains supplementary material available at10.1186/s12882-025-04296-9.

## Introduction

Hemodialysis is the primary modality in Egypt; the use of peritoneal dialysis is currently limited due to the high cost of the treatment and the lack of a national program to promote peritoneal dialysis [1]. Therefore, establishing and maintaining vascular access is crucial in managing end-stage kidney disease (ESKD) patients and is associated with better overall patient outcomes. Arteriovenous fistulas (AVF), arteriovenous grafts (AVG), and central venous catheters (CVC) represent the three types of VA used for HD [2]. AVFs are generally preferred, considering their more prolonged survival, lower frequency of infections and interventions, and lower costs [2, 3]. The creation of AVFs in patients with advanced CKD is recommended prior to the need for maintenance HD to decrease CVC dependence [4]. However, patients with CKD almost present late with complicated disease, which may necessitate urgent initiation of dialysis and placement of CVC shortly before the creation of AVFs. The time needed for AVF maturation may even prolong the use of CVC. Moreover, the time to first AVF cannulation may be further delayed if the AVF requires assisted maturation. Such AVF delays translate into prolonged CVC dependence, with its associated risk of CVC-related bloodstream infections and possibly central vein stenosis [2]. Furthermore, HD patients may be relegated to CVC use when AVF access sites are exhausted [5]. Late creation of VA for HD has been associated with poor outcomes. Patients undergoing VA surgery ≤ 30 days before HD initiation showed the highest increase in all-cause mortality, hospitalization, and healthcare costs compared to those undergoing VA surgery > 180 days before HD initiation [6]. Of note is that VA practice, including AVFs and AVGs, as well as AVF location and maturation time, is substantially variable worldwide. The survival of AVFs following successful first use is largely heterogeneous among different patients and localities and may be affected by both patient factors and dialysis-related practices [5]. Population-level studies to evaluate the trends in the creation of AVFs and AVGs, as well as the survival rates of AVFs and their related predictors and outcomes, are scarce, particularly in the developing world. This study aimed to identify the trends in the use of HD-VA and its associated characteristics and outcomes in a nationwide survey across Egypt.

## Materials and methods

This cross-sectional study was designed to assess patients on maintenance hemodialysis across Egypt and was conducted in 32 hemodialysis units from 11 governorates as a representative sample of the national dialysis population. The 27 Egyptian governorates were first stratified into three regions—Greater Cairo, Lower Egypt, and Upper Egypt. Then, a sample of governorates was randomly selected from each stratum in proportion to regional distribution. Participating dialysis units were then selected within these governorates, and all eligible patients within those units were included. This multistage cluster sampling approach with stratification was used to ensure feasibility while maintaining geographic representation. Data were collected over a one-year period from May 2023 to May 2024. The questionnaire was developed for the study focusing on maintenance HD patients. This research team formulated questions centered around several key themes: (1) patient demographics and clinical characteristics, (2) VA creation and maintenance trends, (3) complications and comorbidities, and (4) healthcare access and quality. An initial draft of the questionnaire was created by a core team of researchers and then reviewed by additional experts, including nephrologists and dialysis nurses, to ensure clarity and comprehensiveness of the content **(Supplementary 1)**. Due to variability in the completeness of hospital records across participating units—particularly in peripheral and satellite centers—a standardized structured electronic Google Form was used to collect data. This ensured consistent and complete recording of key variables such as patient demographics, vascular access history, complications, and healthcare access. While most data were extracted from existing medical records, limited patient interaction was allowed when essential information was missing or unclear, especially in centers with manual or partially digitized systems. Treating physicians administered the questionnaire to patients during regular dialysis sessions, recording responses directly into the online form. The study aimed to explore trends in vascular access among hemodialysis patients across Egypt, and efforts were made to recruit a representative and diverse sample from multiple governorates, covering both Upper and Lower Egypt, to reflect national distribution as much as possible. The study was registered at the Mansoura University Faculty of Medicine Institutional Review Board (IRB) under the approval number R.22.10.1913.R1 and was conducted in accordance with relevant guidelines and regulations. All participants provided informed consent after being fully informed about the study’s purpose and nature.

Responses were compiled and analyzed using the Statistical Package for Social Science (SPSS) version 25 for Windows on personal computers. Qualitative information was described as percentages and numbers. Quantitative information was defined as means [± standard deviation (SD)] for parametric variables or medians (interquartile range; IQR) for non-parametric variables, as suitable. The significance level was considered at 5% (*p* ≤ 0.05). Demographic and clinical data were reported for all respondents, and incomplete surveys (less than 60% complete) were excluded from the final analysis.

## Results

According to the previously mentioned sampling technique, the study included 3582 participants recruited from 32 HD centers across 11 Egyptian governorates. Regional representation included approximately 40% from Upper Egypt and 60% from Lower Egypt. Observed patterns in VA were analyzed in relation to key demographic characteristics including age, gender, residence, years of education, and marital status. Comorbidities and presumed causes of kidney disease were also captured. Among the participants, 66% had hypertension (HTN), which was reported as the primary cause of ESKD in 34%. Approximately 20% of the participants had diabetes, with diabetes attributed as the ESKD cause in 12%. A pattern of smoking was also noted, with nearly 25% being current or former smokers (Table 1).

**Table 1:** Sociodemographic and medical data of the studied patients (*n* = 3582)

Variable	Descriptive statistics
Age, years	53(41–62)
*Gender*:	
Male Female	2084(58.2%) 1498(41.8%)
*Marital status*:	
Married Non-married	2759(77%) 823(23%)
*Residence*:	
Urban Rural	1689(47.2%) 1893(52.8%)
Lower Egypt Upper Egypt	2206(61.6%) 1376(38.4%)
*Governorates*:	
Dakahlia Aswan Alexandria Sohag Assiut Gharbia Ismailia Cairo Portsaid Fayoum Damietta	930(26%) 640(17.9%) 574(16%) 407(11.4%) 329(9.2%) 167(4.7%) 151(4.2%) 137(3.8%) 112(3.1%) 82(2.3%) 53(1.5%)
*Occupation*:	
Has a job No job	662(18.5%) 2920(81.5%)
*Education*:	
Basic education Higher education	2741(76.5%) 841(23.5%)
*Smoking status*:	
Non-smoker Former smoker Current smoker	2668(74.5%) 449(12.5%) 465(13%)
HD duration, months	44(19–84)
DM	654(18.3%)
Duration of DM, years	15(10–22)
HTN	2392(66.8%)
Duration of HTN, years	10(5–15)
*Original kidney disease*:	
HTN Unknown DM Urological GN ADPKD Chronic interstitial nephritis Chronic pyelonephritis Iatrogenic Pregnancy-related Congenital DM&HTN Contrast induced nephropathy Others	1219(34%) 814(22.7%) 430(12%) 294(8.2%) 259(7.2%) 171(4.8%) 118(3.3%) 115(3.2%) 58(1.6%) 37(1%) 25(0.7%) 7(0.2%) 5(0.1%) 30(0.8%)

Regarding pre-ESKD nephrology care, 62% of the participants reported being followed by a nephrologist before HD initiation. Among these, 40% had initiated care immediately before HD, 30% had care for less than six months, and another 30% had nephrology care for more than six months, demonstrating varied exposure to specialist care prior to access planning (Table 2).

**Table 2:** Vascular access data of the studied patients (*n* = 3582)

Variable	Descriptive statistics
*Specialty of the physician followed the patient before HD*:	
Nephrologist Non-nephrologist	2228(62.2) 1354(37.8%)
*Timing of seeing patient by the nephrologist*:	
Immediately before HD Less than 6 months before HD More than 6 months before HD	1417(39.6%) 1009(28.2%) 1156(32.3%)
*Timing of AVF creation*:	
Before HD After HD	1023(28.6%) 2559(71.4%)
Duration between AVF/AVG creation and starting HD when access created before HD, weeks	8(4–16)
Duration between AVF creation and its use, weeks (Average duration of maturation)	6(6–8)
Number of vascular accesses	2(1–3)
*Initial HD access*:	
Temporary catheter Permanent catheter AVG AVF	2861(79.9%) 30(0.8%) 22(0.6%) 669(18.7%)
*Current HD access*:	
Temporary catheter Permanent catheter AVG AVF	182(5.1%) 209(5.8%) 68(1.9%) 3123(87.2%)
*Second HD access: (n* = 2068)	
Temporary catheter Permanent catheter AVG AVF	662(32%) 88(4.3%) 27(1.3%) 1293(62.4%)
*Third HD access*: (*n* = 922)	
Temporary catheter Permanent catheter AVG AVF	321(34.8%) 81(8.8%) 23(2.5%) 497(53.9%)
*Fourth HD access*: (*n* = 466)	
Temporary catheter Permanent catheter AVG AVF	156(33.5%) 47(10.1%) 14(3%) 249(53.4%)
*Site of current AVF*: (*n* = 3123)	
Radiocephalic Brachiocephalic Brachiobasilic	537(17.2%) 1891(60.6%) 695(22.3%)
*Reported causes of failure of AVF: (n* = 352)	
Thrombosis Inflammation/infection Aneurysm Hematoma Hypotension	245(69.6%) 62(17.6%) 18(5.1%) 16(4.5%) 11(3.1%)
Patients with routine examination of vascular access	2632(73.8%)
*Frequency of routine vascular access*: (*n* = 2632)	
Once per week Once biweekly Once monthly Less than once monthly	1268(48.2%) 138(5.2%) 765(29.1%) 461(17.5%)
Patients referred to vascular surgeons	2174(61%)
Routine anti platelet therapy	1380(38.7%)
Routine omega 3 therapy	569(15.9%)

Trends in access timing and type were also explored: more than 70% of the cohort had their AVF created after HD initiation. Among the remaining 30%, the median time between AVF creation and HD start was eight weeks, with the average maturation time ranging between six to eight weeks. A temporal trend in initial access type was noted: 79.9% of participants started HD using non-tunneled central venous catheters (NT-CVC), with tunneled catheters (T-CVC) used in < 1% of cases. The brachiocephalic AVF was the most common (60.6%), followed by brachiobasilic (22.3%) and radiocephalic (17.2%) (Table 2).

Sequential access use trends revealed that AVF served as the first access in 18.7% of patients, second in 36%, third in 13%, and fourth in 6%. Regarding access assessment, 48.2% reported weekly evaluations, while 29.1% had monthly reviews. Antiplatelet thromboprophylaxis was reported in 38.7%. Referral trends showed that 61% of patients had at least one vascular surgical consultation. AVF thrombosis was the leading cause of access failure (69.7%) **(**Table 2**)**.

Geographic and sociodemographic trends in AVF timing were evident: pre-HD AVF creation was more commonly observed in Lower Egypt compared to Upper Egypt (36.3% vs. 16.2%, *P* < 0.001). An earlier AVF placement was noted among patients with higher educational attainment (32.5% vs. 27.4%, *P* = 0.005), and those under nephrologist care compared to primary care (39.1% vs. 11.2%, *P* < 0.001). The duration of nephrology care prior to HD correlated with higher pre-HD AVF proportions. These patterns in care were associated with improved outcomes, including better fistula maturation, fewer VA changes, and reduced access-related complications and surgical referrals (Table 3).

**Table 3:** Timing of AVF creation

	Before HD	After HD	*P* value
**Determinants**			
Age, years	54(41–63)	53(40.8–62)	0.140
*Gender*:			0.590
Male Female	588(28.2%) 435(29%)	1496(71.8%) 1063(71%)	
*Marital status*:			0.536
Married Non-married	795(28.8%) 228(27.7%)	1964(71.2%) 595(72.3%)	
*Residence*:			0.637
Urban Rural	476(28.2%) 547(28.9%)	1213(71.8%) 1346(71.1%)	
*Lower Egypt* *Upper Egypt*	800(36.3%) 223(16.2%)	1406(63.7%) 1153(83.8%)	**< 0.001**
*Occupation*:			0.255
Has a job No job	201(30.4%) 822(28.2%)	461(69.6%) 2098(71.8%)	
*Education*:			**0.005**
Basic education Higher education	750(27.4%) 273(32.5%)	1991(72.6%) 568(67.5%)	
DM	195(29.8%)	459(70.2%)	0.431
HTN	692(28.9%)	1700(71.1%)	0.487
*Specialty of the physician followed the patient before HD*:			**< 0.001**
Nephrologist Non-nephrologists	871(39.1%) 152(11.2%)	1357(60.9%) 1202(88.8%)	
*Timing of seeing patient by the nephrologist before HD*:			**< 0.001**
Immediately before HD Less than 6 months before HD More than 6 months before HD	140(9.9%) 341(33.8%) 542(46.9%)	1277(90.1%) 668(66.2%) 614(53.1%)	
**Consequences**			
Duration between AVF creation and its use, weeks (Average duration of maturation)	7.05(6–12)	6(6–7)	**< 0.001**
Number of vascular accesses	1(1–2)	2(1–3)	**< 0.001**
*Initial HD access*:			**< 0.001**
Temporary catheter Permanent catheter AVG AVF	419(14.6%) 11(36.7%) 16(72.7%) 577(86.2%)	2442(85.4%) 19(63.3%) 92(27.3%) 6(13.8%)	
*Reported causes of failure of vascular access: (n* = 352)			**0.007**
Thrombosis Inflammation/infection Aneurysm Hematoma Hypotension	72(29.4%) 11 (17.7%) 9(50%) 9(56.3%) 2(18.2%)	173(70.6%) 51(82.3%) 9(50%) 7(43.7%) 9(81.8%)	
Patients referred to vascular surgeons	618(28.4%)	1556(71.2%)	0.827

## Discussion

The condition of VA among HD patients is a crucial component of renal care and is linked mainly to the socioeconomic and healthcare system as a whole. VA is of the utmost importance for HD patients, as it affects the treatment efficiency and adequacy. Nevertheless, the condition of VA in Egypt is a multifaceted subject, shaped by a range of obstacles and prospects inherent in the nation’s healthcare infrastructure and primary care. Our study encompassed a robust nationwide cohort of 3582 HD patients across 11 governorates strategically selected to represent a diverse cross-section of Egypt’s geographical landscape. Encompassing both rural and urban areas and spanning across Upper and Lower Egypt, our cohort aimed to capture the breadth of demographic, socioeconomic, and healthcare disparities prevalent within the Egyptian population. By including patients from various governorates, we sought to ensure a comprehensive understanding of VA patterns and challenges that may differ based on regional characteristics and healthcare resource availability. This broad representation enhances the generalizability of our findings and provides valuable insights into the complexities of VA management in the Egyptian HD population. The findings of this study provide a critical opportunity to compare the current state of vascular access in Egypt with international guidelines and identify actionable gaps in care. We meticulously examined a spectrum of variables, including age, gender, residential status, educational attainment, smoking habits, comorbidities such as diabetes, HTN, the underlying etiology of kidney disease, and prevalent cardiovascular morbidities. By scrutinizing these determinants, we aimed to unravel nuanced patterns and identify potential risk factors predisposing individuals to suboptimal VA outcomes. Such insights are paramount for tailoring personalized interventions and refining healthcare strategies to optimize VA care and improve long-term dialysis outcomes in this vulnerable population.

The prevalence of AVF-based HD access has seen significant global attention over the past two decades. In 2003, only 35% of patients with ESKD used AVFs [7], which led to initiatives like the Fistula First Breakthrough Initiative (FFBI) and later the Fistula First Catheter Last (FFCL) program in 2005 [8]. These efforts aimed to increase AVF use to 68% while reducing long-term catheter use to less than 10%. This shift was accompanied by comprehensive programs to promote early planning and improve patient quality of life. The Dialysis Outcomes and Practice Patterns Study (DOPPS) further illustrated this trend: AVF use in the U.S. increased from 24% in DOPPS I to 68% in DOPPS IV and V. Meanwhile, catheter use declined from 17 to 15%. However, the percentage of patients initiating HD with a catheter remained high at 67% [9–14].

In the present data, 79.9% of Egyptian patients still begin HD with NT-CVC, while 18.7% use AVF as initial access, indicating an improvement since Afifi’s 2002 study [15], where 90% required temporary access and only 10.2% had a fistula created before dialysis initiation. This modest progress may be limited by patient refusal and less strict adherence to timely referrals to VA establishment. In addition, in the current study, patients who received nephrology care were more likely to have AVF as a primary access (25.5%) ompared to non-specialized nephrology care by an internist or a general practitioner (7%). The use of NT-CVCs to initiate HD is found to be significantly lower in patients who had received nephrology care for more than 6 months before diagnosis of ESKD compared to patients with delayed referral (26% vs. 46% respectively). These numbers harmonized with the United States Renal Data System report in 2018, which showed that using non-tunneled lines as a primary VA occurred in 80.8% of ESKD patients compared to 16.2% and 3% for AVF and AVG, respectively. Nevertheless, in patients who had an early nephrology care for more than 12 months before the diagnosis of ESKD, initial AVF use was reported in 29.7%. Of note, prevalent rather than incident HD patients were more likely to use AVF (65.7%), followed by CVCs and AVGs in 17.6% and 16.7%, respectively [13]. Also, in a Spanish study, the timing of referral to nephrology care was shown to significantly influence vascular access outcomes [16].

High CVC use is a globally reported problem. A previous study from the UK showed that approximately one-third of patients presented late, thus requiring CVCs to be used as primary access [17]. An extensive US survey revealed that out of 110,000 incident HD patients annually, only 20% start dialysis with an AVF or AVG, whereas the remaining 80% start with a CVC [11]. According to DOPPS, reasons to utilize a CVC rather than an AVF at dialysis initiation were lack of proper vessels and limited life expectancy, followed by patient refusal for AVF creation [13]. Interestingly, patient refusal for AVF creation represented a valid reason to utilize CVC in 71% of cases in an earlier Canadian study. They suggested that patients may favor a CVC rather than AV access for several reasons (fear of needling, dislike of the appearance of AVFs, arm discomfort during dialysis, etc [18]. The present study highlighted the late referral patterns among Egyptian patients, with about 70% of patients being referred to a nephrologist in less than 6 months of starting HD. Although 28.5% of the included patients had AVF access created before starting HD, only 19.5% had mature AVF. This might be attributed to late referrals to vascular surgeons and patient factors contributing to delayed maturation. It is clear that such patients do not rapidly move towards definitive access in a timely fashion. Secondary to this, late referral of about two-thirds (71.4%) of patients causes delayed AVF creation after the initiation of HD.

For this, KDOQI guidelines recommended that patients with CKD should be referred to nephrologists to begin discussions about modality choices when the estimated glomerular filtration rate is less than or equal to 30 mL/min. Nephrologists should refer patients to vascular surgeons/ interventionalists for VA assessment and creation when their estimated glomerular filtration rate is 15 to 20 mL/min with concurrent progressive decline in kidney function or earlier if they have a rapid decline in estimated glomerular filtration rate (> 10 mL/min/y). Moreover, they emphasized a patient-centered approach that considers crucial issues such as comorbidities, patient goals, preferences, financial and social support, and logistics of HD to choose the optimal VA for HD patients [19]. Failure to establish timely functioning permanent VA poses significant implications on patient survival. HD initiation with mature permanent access leads to better outcomes compared to patients starting with an HD catheter [20].

Our study revealed a significant deviation from the international practices and guidelines regarding using T-CVCs for temporary VA. According to guidelines, T-CVCs are recommended over NT-CVCs when access is required for more than two weeks, as they are associated with fewer complications, including infection and venous stenosis [18]. However, in our cohort, less than 1% of patients had T-CVCs placed. This stark contrast is primarily attributed to the lack of specialized paraphernalia for T-CVC insertion and associated high costs, particularly in peripheral dialysis units.

The patterns of the VA creation site show notable global variations. Our findings align with some but not all international trends. In our study, upper-arm AVFs were the most commonly used (83%), while 13% of patients had lower-arm AVFs. This contrasts with the reported high use of lower-arm AVFs in Japan, although the use of lower-arm AVFs has been significantly declining in the US—from 70% in the early DOPPS phases to 32% more recently. Europe maintains intermediate use, though the lower-arm AVF prevalence has also decreased over time. These regional differences are influenced by multiple factors, including dialysis vintage, diabetes, and peripheral vascular disease, as noted in various international studies [5].

The predominance of upper-arm AVFs in our data, specifically brachiocephalic (60.6%) and brachiobasilic (22.3%) sites, suggests a trend similar to regions where late ESKD diagnosis, late referrals, diabetes, and vascular complications are common. Although we did not directly test these predictors, these factors may explain our cohort’s high use of upper-arm AVF. This trend warrants further investigation, particularly regarding younger HD patients, as early use of upper-arm sites limits future AVF options. Upper-arm AVFs are associated with a higher risk of steal syndrome and potential long-term cardiovascular effects due to increased blood flow [21–23]. It is worth mentioning that the 2002 Egyptian report [15] noted the radiocephalic arteriovenous fistula as the most prevalent type, accounting for 67.3% of studied cases. In light of these findings, it is essential to highlight that the latest KDOQI 2019 guidelines [19] recommend distal AVF placement whenever feasible, as distal fistulas are associated with fewer complications and preserve more proximal sites for future use. Tailoring VA strategies to individual patient’s clinical circumstances is crucial, and future initiatives should aim to improve early referral and address barriers to timely lower-arm AVF placement.

In our study, 38% of patients undergoing AVF creation were prescribed antiplatelet therapy as thromboprophylaxis. This aligns with a broader trend in clinical practice but contrasts with some findings in the literature. A meta-analysis on the use of antiplatelet agents in VA for HD revealed little to no effect on the patency of grafts and uncertain impacts on AVF maturation despite the known antiplatelet protective effects against thrombosis. Moreover, it raised concerns about the risk of significant bleeding. Given the complexities surrounding VA, the meta-analysis highlights the need for interventions that improve access suitability for dialysis [24]. The 2019 KDOQI guidelines reflect these uncertainties, with inadequate evidence supporting a formal recommendation on the routine use of antiplatelets to enhance AVF usability or patency [19].

In our study, 73% of patients underwent routine surveillance for AVF complications, highlighting an opportunity for improvement. Recent guidelines emphasize the importance of regular clinical evaluations to maintain VA health [19]. However, it is noteworthy that the approach aligned with the current recommendations, which advocate for ongoing clinical assessments for those routinely examined. Radiological evaluations and vascular surgeon referrals were appropriately utilized when complications occurred.

A limitation of our study is its reliance on questionnaire-based data, which inherently limits the depth of specific details. For instance, we could not fully explore the relationship between early and late complications and specific patient characteristics, such as the impact of underlying kidney disease or comorbid conditions. Additionally, we could not assess the patients’ outcomes concerning the reported AVF failures due to complications like thrombosis or bleeding. These factors may have provided a more comprehensive understanding of the causes and impacts of VA complications. Third, the cross-sectional nature of the study limits the ability to infer causality or evaluate temporal changes in vascular access practices over time. Instead, the study presents distributional and practice trends across different demographic and regional strata at a single time point. Finally, while a tailored data collection tool was developed to address the specific objectives of this study, we acknowledge that it was not formally validated through standard psychometric methods such as Cronbach’s alpha. This was primarily due to the absence of a suitable pre-existing instrument that comprehensively addressed the clinical and health system dimensions relevant to our context.

In conclusion, our study highlights the significant variations in VA practices for HD patients in Egypt, aligning with some global trends while diverging from others. Addressing these issues of late referral to nephrologists, low initial AVF usage, and high NT-CVC rates requires a multifaceted approach. Implementing educational programs for primary care providers and general practitioners is a critical first step to ensure early referral to nephrologists, as recommended by the Kidney Disease Outcomes Quality Initiative (KDOQI) guidelines. This can facilitate timely AVF creation and maturation, improving the rates of starting with AVF. To reduce the utilization of NT-CVCs, it is essential to address patient factors, such as fears and misconceptions about AVF. This could be achieved through structured patient education programs that address concerns about AVF appearance, needling fear, and discomfort. As for the implementation of T-CVC strategies, there’s a pressing need to equip dialysis units with the required resources for T-CVC insertion. This includes not only the specialized equipment but also training for the healthcare professionals involved. Additionally, addressing financial constraints through governmental or non-governmental support programs can also facilitate the more frequent use of T-CVCs, thereby aligning the practice in Egypt with the international guidelines.

## Electronic supplementary material

Below is the link to the electronic supplementary material.

Supplementary Material 1

## Data Availability

The data supporting the findings of this study are available from the corresponding author upon reasonable request.

## Abbreviations

AVF: Arteriovenous fistula
AVG: Arteriovenous graft
CKD: Chronic kidney disease
CVC: Central venous catheters
DOPPS: Dialysis Outcomes and Practice Patterns Study
ESKD: End-stage kidney disease
FFCL: The Fistula First Catheter Last
HD: Hemodialysis
HTN: Hypertension
IQR: Interquartile range
IRB: Institutional Review Board
KDOQI: Kidney Disease Outcomes Quality Initiative
NT-CVC: Non-tunneled central venous catheter
SD: Standard deviation
SPSS: Statistical Package for Social Science
T-CVC: Tunneled central venous catheter
VA: Vascular access
