# Supplementary material for: Trends in vascular access among patients on hemodialysis; a nationwide survey from Egypt
Source: BMC Nephrol. 2025 Jul 8;26:361. doi: 10.1186/s12882-025-04296-9 (PMC12235907; doi:10.1186/s12882-025-04296-9)
Supplement: Supplementary file 1 — Supplementary Material 1 [file 12882_2025_4296_MOESM1_ESM.pdf]

Vascular access survey

1. Name of the clinic
2.Name of the patient
3.Age
4.Gender
5.Marital status
6.Number of children
7.Residence
8.Governorate
9.Work
10.If the patient has a job, specify
11.Educational level *
12.Smoking status
13.Other special habits
14.Date of starting HD
15.Cause of ESKD
16.DM
17.Duration of DM (years)
18.HTN
19.Duration of HTN (years)
20.Have you been previously informed about PD *
21.Initial HD access
22.When he was advised for AVF creation?
23.Specialty of the physician who was following the patient before HD
24.When was the patient seen by the nephrologist before HD?
25.Duration in weeks between AVF/AVG creation and starting HD (If AVF created before HD)
26.Duration in weeks between starting HD and AVF/AVG creation (If AVF created after HD)
27.Duration of days (average) between starting HD and use of AVF/AVG
28.Duration in weeks between AVF creation and its use
29.Current vascular access
30.Number of vascular accesses
31.Type of second vascular access
32.Date of second vascular access
33.Cause of change to second vascular access
34.Type of third vascular access
35.Date of third vascular access
36.Cause of change to third vascular access
37.Type of fourth vascular access
38.Date of fourth vascular access
39.Cause of change to fourth vascular access
40.Any routine examination in HD units for the Access
41.If the answer to the previous question is yes, how frequent the examination is done? *
42.Referral to vascular surgeon
43.Routine anti platelet therapy
44.Routine omega 3 therapy
45.Site of AVF
